# Genital rash: A manifestation of metastatic Crohn's disease

**DOI:** 10.1002/ccr3.9314

**Published:** 2024-08-20

**Authors:** John David Chetwood, Arteen Arzivian, Joo‐Shik Shin, Aravind Gokul Tamilarasan

**Affiliations:** ^1^ Concord Repatriation General Hospital Concord New South Wales Australia; ^2^ AW Morrow Gastroenterology and Liver Centre Royal Prince Alfred Hospital Sydney New South Wales Australia; ^3^ Sydney Medical School University of Sydney Sydney New South Wales Australia; ^4^ Saint Vincent's Hospital Darlinghurst New South Wales Australia; ^5^ Faculty of Medicine, Health and Human Sciences, Macquarie Medical School Macquarie University Sydney New South Wales Australia; ^6^ Department of Pathology Royal Prince Alfred Hospital Sydney New South Wales Australia

**Keywords:** case report, Crohn's disease, cutaneous, extra‐intestinal manifestations, infliximab

## Abstract

Metastatic Crohn's disease is the rarest cutaneous manifestation of Crohn's disease, it presents as cutaneous lesions in areas that are anatomically non‐contiguous with the gastrointestinal tract. It requires a high index of suspicion for diagnosis which is confirmed on histopathology. Infliximab can be an effective treatment.

## 1 INTRODUCTION

Mucocutaneous lesions are the most common extra‐intestinal manifestation (EIM) of Crohn's disease (CD). It is estimated that more than 10% of patients with inflammatory bowel diseases (IBD) will present with a mucocutaneous EIM, most commonly erythema nodosum, pyoderma gangrenosum, and aphthous stomatitis.^1^ Skin involvement in CD can be classified into five groups: (1) Specific manifestations that share the same histological features as CD, for example, peri‐anal CD or metastatic CD (MCD), a rare manifestation, (2) Skin disorders associated with but do not have mutual pathogenic or histological features as CD, for example, aphthous stomatitis and psoriasis, (3) Reactive mucocutaneous disorders that share similar pathogenic mechanisms with CD but differ histopathologically, for example, pyoderma gangrenosum, (4) Adverse mucocutaneous reaction to treatments used for CD, most frequently associated with anti‐tumor necrosis factor agents, (5) mucocutaneous manifestations due to nutritional deficiencies.^1^, ^2^ Most of these lesions can be diagnosed with certainty based on their appearance and clinical characteristics. However, atypical presentations of common lesions or lesions with rare occurrences remain a diagnostic challenge that requires a biopsy and dermatological review.^2^ We describe herein a case of MCD and our workup and treatment of this rare cutaneous manifestation.

## 2 CASE HISTORY/EXAMINATION

**FIGURE 1:**
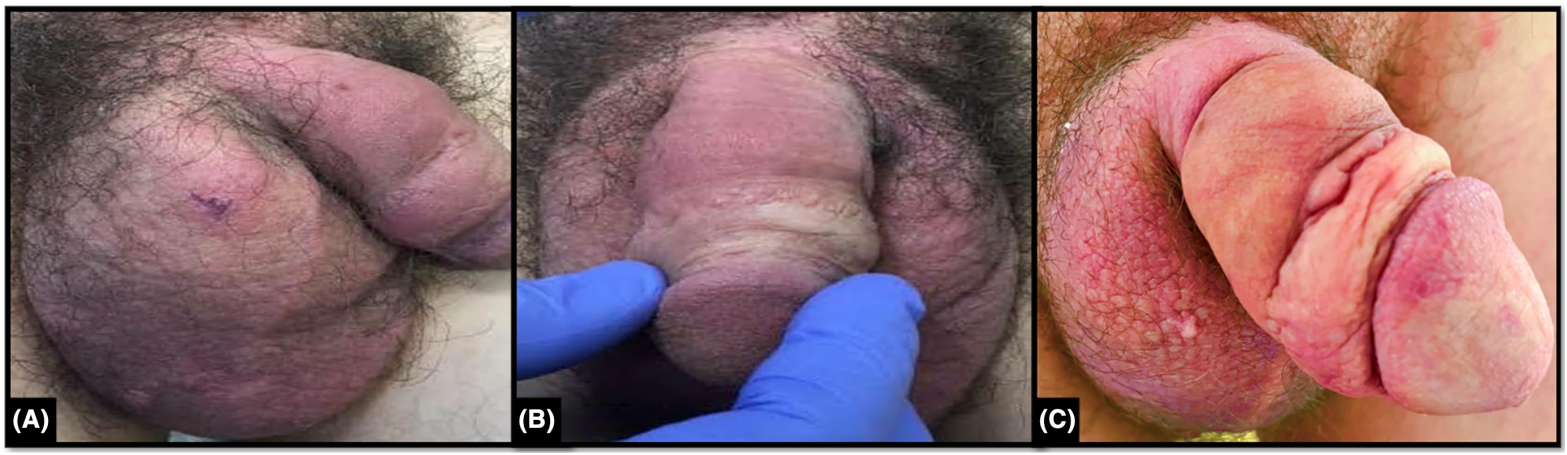
(A–C) Metastatic Crohn's disease. Macroscopic appearance of the genital region at index, with markedly oedematous violaceous cutaneous plaques in the scrotum and penis.

A 34‐year‐old man presented with a 2‐month history of a painful rash affecting the intertriginous groin, scrotum, and penile body, causing painful erectile dysfunction (Figure 1). Physical examination revealed confluent oedematous violaceous plaques with no other cutaneous sites affected. His medical history included perianal and ileocolonic Crohn's disease, and at the index presentation, he was in clinical remission (Harvey‐Bradshaw index 1) on vedolizumab at standard dosing (300 mg intravenously 8‐weekly).

## 3 METHODS (DIFFERENTIAL DIAGNOSIS, INVESTIGATIONS, AND TREATMENT)

**FIGURE 2:**
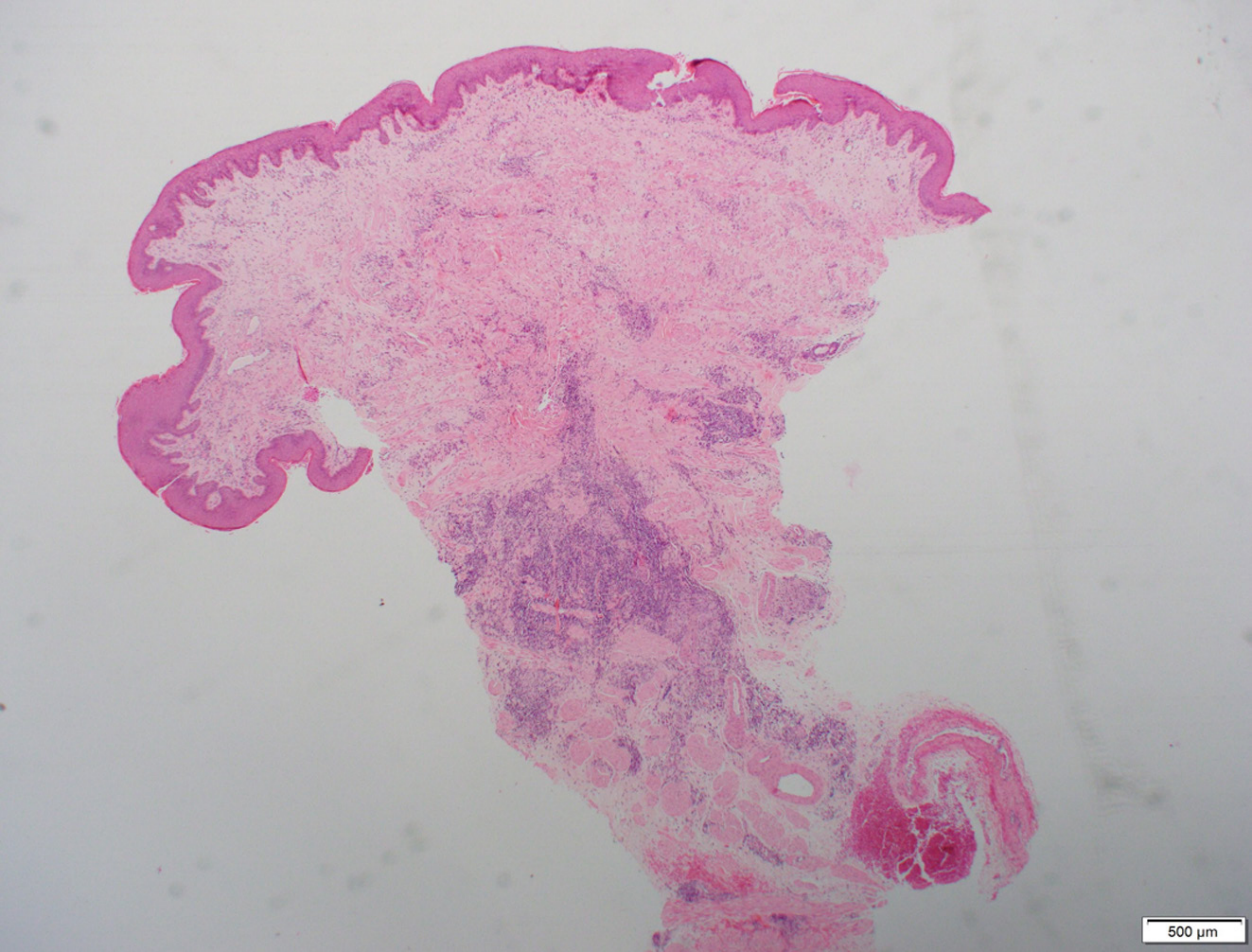
Metastatic Crohn's disease. A scrotal skin biopsy with hematoxylin–eosin staining on a low‐power field view (×20) demonstrates a dense cellular infiltrate centred in the deep dermis without significant epidermal involvement.

**FIGURE 3:**
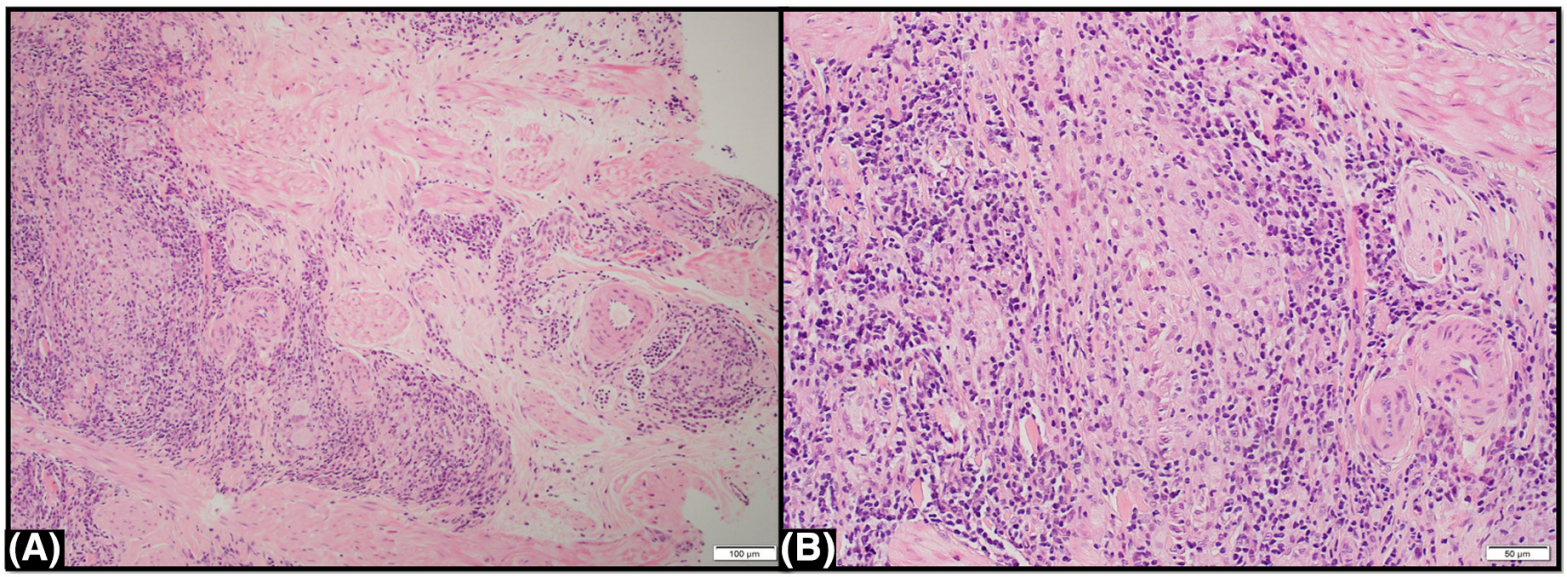
(A, B) Metastatic Crohn's disease. Scrotal skin biopsy with hematoxylin‐eosin staining on high power field view (×200), demonstrating the non‐caseating granulomas with histiocytic aggregates surrounded by dense lymphocytes and plasma cells.

Scrotal skin biopsy showed non‐caseating granulomatous aggregates with epithelioid multinucleated histiocytes, lymphocytes and plasma cells without evidence of fungal organisms, acid‐fast bacilli, or vasculitis (Figures 2 and 3). The clinical findings and histology were consistent with a diagnosis of anogenital MCD.

Prior treatments for Crohn's disease had included adalimumab (used in combination with azathioprine) with secondary loss of clinical efficacy and ustekinumab, which resulted in adverse drug reaction (anaphylaxis). Vedolizumab was ceased, and the patient was treated with dose‐intensified infliximab, azathioprine, and topical betamethasone. Therapeutic drug monitoring (TDM) was utilized to ensure adequate infliximab and azathioprine dosing. The infliximab trough level on 5 mg/kg 8‐weekly was 14.0 mg/L; however, in the context of the active disease and disease‐resistant cutaneous presentation, infliximab therapy was dose‐escalated to 4‐weekly, which achieved a slightly higher infliximab trough level of 16.3 mg/L. Topical tacrolimus and triamcinolone acetonide were initially trialed but ceased due to limited efficacy.

## 4 CONCLUSION AND RESULTS (OUTCOME AND FOLLOW‐UP)

This combined approach led to a marked improvement in the cutaneous manifestations and resolution of his painful erectile dysfunction after 10 months of therapy.

## 5 DISCUSSION

MCD was first described in 1965 by Parks et al. as a “sarcoid reaction” on biopsy in two individual cases.^3^ The diagnosis relies on the presence of Crohn's disease histopathological characteristics, for example, non‐caseating granuloma in areas of the skin that are non‐contiguous with the gastrointestinal tract. The limbs are most commonly affected in adults, followed by the genitalia (anogenital Crohn's disease) and face.^3^

Histologically, MCD is typically characterized by non‐caseating granulomatous inflammation with the presence of perivascular monocytes and lymphocytes in the superficial dermis, deep dermis and adipose tissue.^4^, ^5^, ^6^ Associated features may also include eosinophilic infiltrate, collagen degeneration, oedema and ulceration of the overlying epidermis.^7^ Less common patterns of MCD include lichenoid and granulomatous dermatitis encroaching on the epidermis, with granulomatous vasculitis also being described.^8^, ^9^, ^10^ The granulomas in intestinal and dermal tissue should share the same macroscopic appearance in MCD.^6^ Cutaneous findings of MCD vary with age; lymphoedema is more common in pediatric cases, whilst nodules, ulceration, and fistulizing disease are more common in adults.^11^ 85% of pediatric MCD involve the genitals, compared with 23% in adults.^6^ Females are twice as commonly affected by MCD than males in the reported literature to date.^6^, ^7^, ^12^

The differential diagnosis of MCD includes IBD and non‐IBD‐related pathology. The macroscopic appearance of MCD may be challenging to differentiate from more common cutaneous manifestations of CD. Pyoderma gangrenosum typically presents as a nodule on the extensor surfaces that rapidly develops into a deep ulcer and can be identified histologically by pseudoepitheliomatous hyperplasia and copious neutrophilic infiltrate^6^, ^13^ Erythema nodosum manifests as tender, inflammatory, subcutaneous nodules on extensor surfaces of lower extremities that correlate with intestinal disease activity. A biopsy may cause aggravation of erythema nodosum and is generally not recommended.^14^ Other differentials for MCD include sarcoidosis, tuberculosis, granulomatous cheilitis, connective tissue disorders and fungal infections—which should be ruled out prior to immunosuppressive treatment.^15^ In comparison to sarcoidosis histologically, granulomata of Crohn's are less defined, ulceration and oedema are more common, and eosinophils are more prominent.^7^ Ziehl‐Neelsen staining with polymerase chain reaction and tuberculosis culture is often required to exclude *Mycobacterium tuberculosis*, and serology with histopathological staining for auto‐immune and infective pathologies may also be required.

Anogenital MCD is extremely rare; only 410 cases have been reported worldwide.^3^, ^16^ Infliximab is most commonly reported for treatment in 30% of published cases, achieving clinical remission in just 32.5%.^1^ Alternate agents include steroids, antibiotics, tacrolimus, thiopurines, and, more rarely, ustekinumab and hyperbaric oxygen.^3^ TDM is often recommended despite the paucity of available evidence, as high serum infliximab levels have shown a therapeutic correlation with other disease‐refractory subtypes, such as fistulising CD.^16^, ^17^ However, to date, there have been no adult cases reported where anogenital MCD has been treated with TDM, though it was previously described in one pediatric patient.^18^

In conclusion, we present a rare cutaneous manifestation of anogenital MCD and its subsequent identification and treatment using TDM. It is clinically important to distinguish cutaneous manifestations of Crohn's disease because each entails different sequelae and treatment regimens.

## AUTHOR CONTRIBUTIONS

**John David Chetwood:** Conceptualization; formal analysis; methodology; project administration; supervision; writing – original draft; writing – review and editing. **Arteen Arzivian:** Conceptualization; data curation; methodology; software; writing – original draft. **Joo‐Shik Shin:** Conceptualization; supervision; validation; writing – review and editing. **Aravind Gokul Tamilarasan:** Conceptualization; methodology; project administration; supervision; validation; writing – review and editing.

## FUNDING INFORMATION

None.

## CONFLICT OF INTEREST STATEMENT

The authors declare no conflicts of interest.

## CONSENT

Written informed consent was obtained from the patient to publish this report in accordance with the journal's patient consent policy.

## Data Availability

The data supporting this study's findings are available upon reasonable request from the corresponding author. The data are not publicly available due to privacy and ethical restrictions.
